# Artificial light and biting flies: the parallel development of attractive light traps and unattractive domestic lights

**DOI:** 10.1186/s13071-020-04530-3

**Published:** 2021-01-07

**Authors:** Roksana Wilson, Andrew Wakefield, Nicholas Roberts, Gareth Jones

**Affiliations:** School of Biological Sciences, University of Bristol, Life Sciences Building, 24 Tyndall Avenue, Bristol, BS8 1TQ UK

**Keywords:** Diptera, Light attraction, Phototaxis, Spectral wavelength preferences, Vector

## Abstract

Light trapping is an important tool for monitoring insect populations. This is especially true for biting Diptera, where light traps play a crucial role in disease surveillance by tracking the presence and abundance of vector species. Physiological and behavioural data have been instrumental in identifying factors that influence dipteran phototaxis and have spurred the development of more effective light traps. However, the development of less attractive domestic lights has received comparatively little interest but could be important for reducing interactions between humans and vector insects, with consequences for reducing disease transmission. Here, we discuss how dipteran eyes respond to light and the factors influencing positive phototaxis, and conclude by identifying key areas for further research. In addition, we include a synthesis of attractive and unattractive wavelengths for a number of vector species. A more comprehensive understanding of how Diptera perceive and respond to light would allow for more efficient vector sampling as well as potentially limiting the risk posed by domestic lighting.
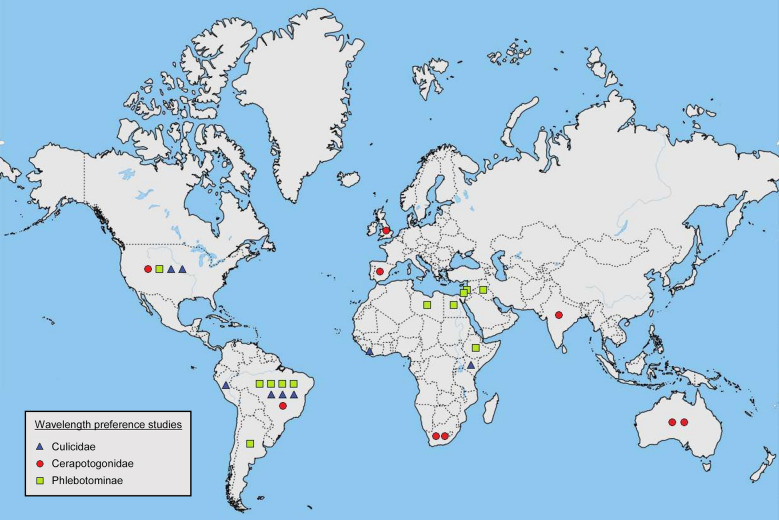

## Background

Haematophagy (blood-feeding) has evolved independently multiple times amongst the Diptera [1]. Haematophagous flies are collectively known as ‘the biting flies’, and their lifestyle facilitates the transmission of blood-borne pathogens from one host animal to another. To humans, the most dangerous and prolific of this group are the mosquitoes (Culicidae), which cause hundreds of thousands of deaths annually through the transmission of pathogens such as malaria, dengue and yellow fever [2]. Other vectors include sand flies (Phlebotominae), black flies (Simuliidae) and tsetse flies (Glossinidae), which transmit pathogens that cause leishmaniasis, river-blindness and African sleeping sickness, respectively—causing disfigurement, disability and chronic suffering [2]. Biting midges (Ceratopogonidae) play a limited role in the transmission of pathogens that cause disease in humans but cause considerable economic impact by spreading bluetongue virus and pathogens causing African horse sickness amongst livestock [3]. These diseases disproportionately affect the poorest populations, with deaths being highest in African countries [4].

The abundance and distribution of biting fly populations should be closely monitored so that the risk of disease outbreak can be determined and the effectiveness of vector-control strategies evaluated. Light traps have been criticised for their bias towards certain taxa and flies of a certain parity status (particularly human-feeding, host-seeking females), and catches can be unrepresentative of the local population [5–8]. However, due to their widespread availability, ease of use, lack of risk to collectors from infectious flies and minimal influence from human error, they are now routinely used in the capture of mosquitoes [9], midges [10] and sand flies [11]. Attention has since shifted to the development of more attractive light traps [12–16]. Highly attractive lights are more likely to detect rare vector species, or increase the capture of sparsely distributed individuals, which allows for control measures to be implemented when the risk of disease outbreak is still low [17–19]. Larger catches also increase the chances of finding infected flies, allowing for virus isolation [18].

Despite the interest in light attraction, the role artificial lights play in facilitating disease transmission by attracting vectors remains understudied [20]. The only definitive example of this phenomenon so far is Chagas disease, spread by triatomine bugs (Order: Hemiptera). Proximity to street lights is linked to house infestation by triatomines [21, 22], and a 2005 disease outbreak was traced to sugarcane juice contaminated with triatomines attracted to the lamp above the juice kiosk [20]. For diseases transmitted by dipterans, correlations between electrification and malaria have been reported in the Solomon Islands [23], Burkina Faso [24], Uganda [25] and Malawi [26]. However, determining whether these outbreaks are caused by artificial lights attracting mosquitoes to human settlements requires further study. Even so, as biting flies are attracted to light, the development of less attractive domestic lights is of considerable importance.

The aim of this review is to outline the ways artificial lights can be made more or less attractive to mosquitoes, midges and sand flies and to identify areas where further research is needed. By modifying light traps and domestic lights, trapping efficiency for vectors could be improved and the public health risk posed by electrification could be reduced.

## Physiology of the dipteran eye

Neurophysiological studies on the visual systems of flies provide a better understanding of light attraction as they reveal sensitivity to different wavelengths of light. However, studies on biting flies are rare [27].

**Fig. 1:**
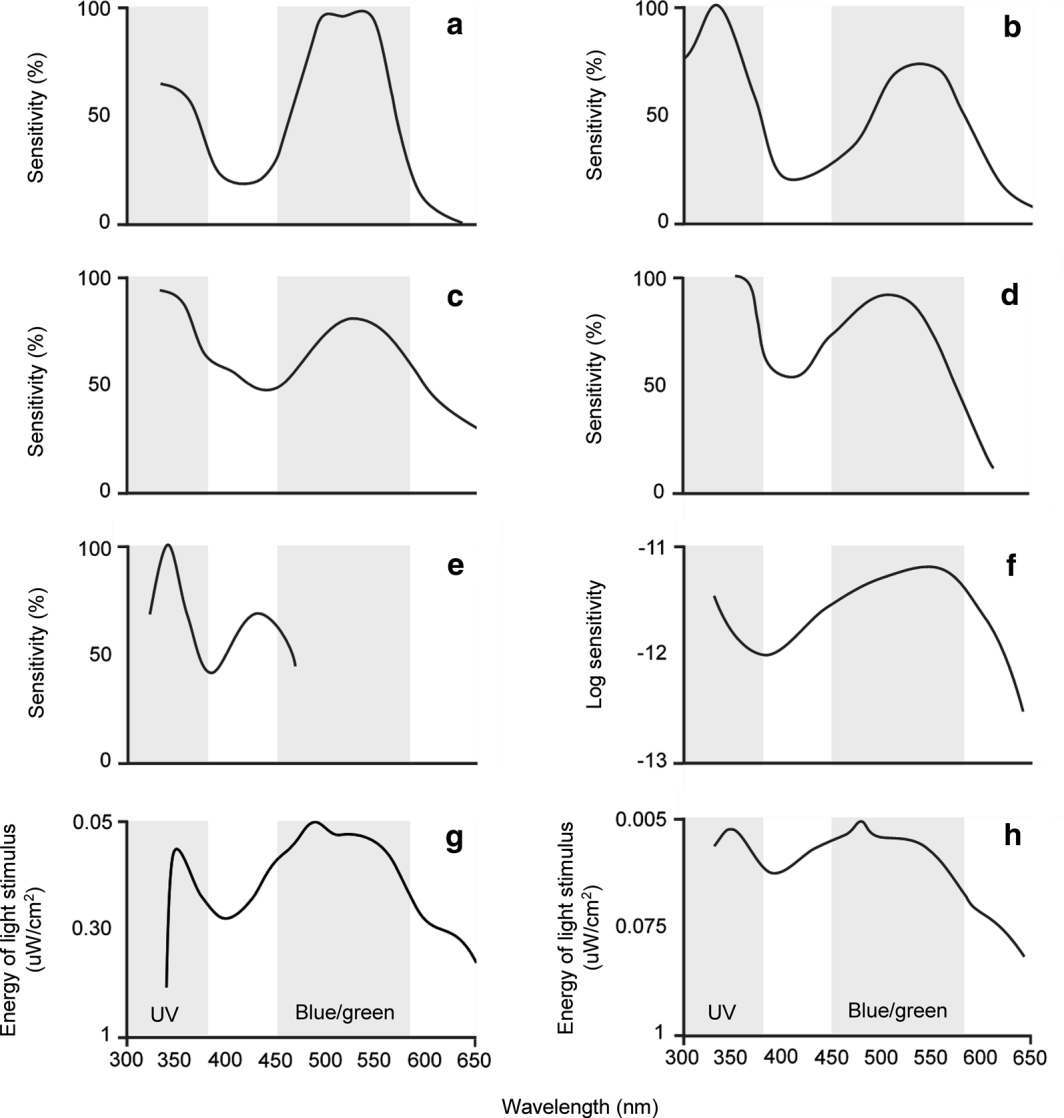
Electroretinograms (ERGs) showing the spectral sensitivities of **a** female *Aedes aegypti* [31], **b** female *Culex pipiens* [32], **c** female *Lutzomyia longipalpis* [33], **d** *Glossina morsitans* [34], **e** male Simuliid blackflies [36], **f** young, female *Tabanus nigrovittatus *[35], **g** *Stomoxys calcitrans* [37] and **h** female *Haematobia irritans* [37]. Figure is adapted from original publications [31–37]. Studies differ in their methods and specimens (age, sex, chromatic adaptation, etc). Ultraviolet and blue/green wavelengths are highlighted in grey

Ommatidia, the units making up the compound eye, contain eight photoreceptor cells known as retinula cells (labelled R1–R8). The spectral sensitivity of these cells, i.e. which wavelength bands the receptor absorbs, depends mainly on the visual pigment rhodopsin (Rh) within each photoreceptor [28]. In *Drosophila*, the R1–R6 cells express Rh1, which responds to a broad spectrum of light, and thus these cells are believed to be achromatic. The R7 cell expresses Rh3 or Rh4 pigments (both ultraviolet [UV] sensitive) and the R8 cell expresses Rh5 (blue sensitive) or Rh6 (green sensitive) pigments, and these cells are assumed to be chromatic [29]. This UV/blue/green sensitivity is highly conserved in insects [30]. For biting flies, spectral sensitivity data exist for *Aedes aegypti* [31], *Culex pipiens* [32], *Lutzomyia longipalpis* [33], *Glossina morsitans* [34], *Tabanus nigrovittatus* [35], simuliid blackflies (species not provided) [36], *Stomoxys calcitrans* [37] and *Haematobia irritans* [37]. These taxa all show dual peaks in sensitivity, with one peak in the UV and another in the blue/green, and minimal sensitivity to longer wavelengths (Fig. 1).

In insects, attraction to specific wavelength bands is controlled by photoreceptors and post-receptor mechanisms. Wild-type *Drosophila* prefer UV light over blue and green wavelengths. However, blocking the activity of Dm8 neurons causes the flies to prefer green light (525 nm) over UV (370 nm) [29]. Dm8 neurons are wide-field amacrine cells located in the medulla. They are the post-synaptic targets of the UV-sensitive R7 cells and provide lateral connections to neurons that project to higher visual centres [29]. Similarly, silencing the R1–R6 cells or the R7 and R8 cells causes flies to prefer blue (430 nm) over UV (350 nm) [38]. Wild-type *Drosophila* also prefer blue wavelengths over green (565 nm), but inactivating the blue-sensitive Rh5, or removing the UV-sensitive R7 cells, causes flies to prefer green [38]. The attractiveness of specific wavelength bands can also vary throughout the day and appears to be circadian regulated [39, 40]. Wild-type* Drosophila* show a peak of UV (365 nm) and blue (460 nm) light avoidance behaviour during midday. However, mutant flies lacking in cryptochrome (cry), the primary circadian light sensor in *Drosophila*, exhibit a strong attraction to UV and blue light at all times of the day [39]. These null cry flies also show an increased attraction to orange (595 nm) light compared to wild-type control flies [39]. An in-depth outline of the mechanisms underpinning phototaxis is beyond the scope of this review, but species-specific differences in wavelength preferences are likely a result of subtle differences in neurophysiology.

Attraction to certain wavelengths is not necessarily a sign of colour vision. True colour vision is the ability to distinguish “light of different spectral compositions (hues) independently of their intensities” [29]. It requires at least two photoreceptors with different sensitivities, and the neural framework to compare the outputs of these receptors [41]. Flies possess at least two photoreceptor types and so fulfil the first precondition of colour vision. However, it is disputed whether insects adapted to low light conditions, which are those most attracted by light traps, would possess colour vision [42]. There are subtle differences between the eyes of nocturnal and diurnal flies, such as whether the eye is configured to increase image resolution at the expense of light sensitivity (apposition eye), increase light sensitivity at the expense of resolution (optical superposition)—or a combination of the two (neural superposition) [43]. These differences may affect how the fly perceives colour.

## Factors that influence light attraction

### Lighting technology

**Fig. 2:**
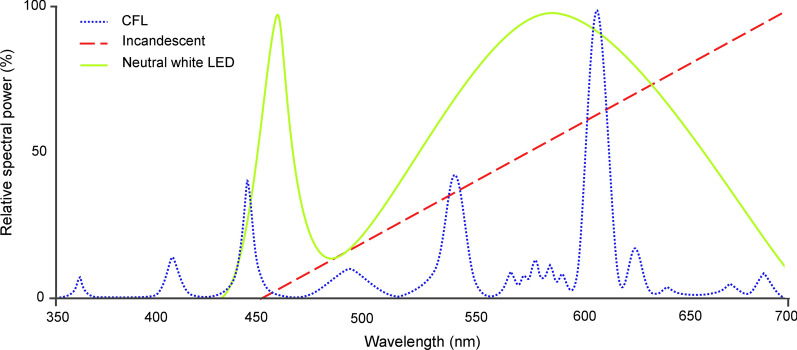
Spectral distribution of three light types: incandescent, compact fluorescent (*CFL*) and neutral-white, light-emitting diode (*LED*). Figure adapted from [116]

There are three main types of lighting technology available to consumers for home use: incandescent; fluorescent; and light-emitting diode (LED) (Fig. 2). The earliest light bulbs were incandescent, and their glow is the result of a wire filament being heated, although most of the electrical current passing through the bulb is emitted as infrared (IR) radiation (heat). Numerous countries have issued bans on these bulbs due to this extreme energy inefficiency [44]. Compact fluorescent lamps (CFLs) became an ‘energy-saving’ alternative to the incandescent bulb. These lamps use mercury to produce UV light, which is then converted into visible light when it strikes the fluorescent phosphor coating on the inside of the bulb. The amount of UV light converted depends on the design or quality of the lamp, and as mercury is a bioaccumulating pollutant, CFLs are classified as hazardous waste during disposal. The newest technology are the LEDs: semiconductors that emit light when a current is passed through them. LEDs have many advantages over other light types, including improved energy efficiency, low power consumption, longer lifetime, high durability, cheaper cost and the ability to produce monochromatic light in a variety of wavelengths [16].

The global market share of LED lamps was approximately 36% in 2015 and is predicted to reach 67–80% in 2022. The highest growth rates are expected in Africa, Asia-Pacific, India and Latin America [45]. These are regions with high death rates from vector-borne disease [4], making it important to determine how these lights perform as vector attractants. A small difference in attractiveness between two lights can become a large difference in insect numbers when many lights are used over a wide area. Conversely, LEDs could represent a cheap, easy and highly effective surveillance tool.

Light traps using incandescent bulbs generally catch fewer flies than light traps emitting UV (<400 nm) light [46–52]. However, LEDs can be both less attractive [9, 15, 17, 49, 53–57] and more attractive [18, 19, 58–63] than incandescent or fluorescent lights. In studies where LEDs have been found to be less attractive, the LED is usually white. Typically, white LEDs operate by converting almost all of the electrical energy they receive into light in the visible spectrum. This means they lack the IR (> 700 nm) and UV peaks found in incandescent and fluorescent lights, respectively (Fig. 2). UV light has been characterised as attractive to insects, and high amounts of IR radiation could act as a thermal attractant alongside the light [53, 56]. In contrast, LEDs emitting narrow bands of short wavelength light generally attract more insects than the broad-spectrum incandescent and fluorescent lights. As LED arrays are highly malleable with regard to spectral composition, they can be tailored to reduce or increase insect catches depending on the need.

### Wavelength

In comparisons of lamps emitting narrow wavelengths of light, mosquitoes, *Culicoides* midges and sand flies have generally been found to be attracted in higher numbers by short wavelengths, such as UV, blue (450–495 nm) and green (495–570 nm) light [17–19, 46, 50, 52, 58–70]. A UV light trap emitting predominantly at 325 nm caught fewer mosquitoes than UV traps emitting at 350–365 nm [50], possibly suggesting that shorter UV wavelengths are less attractive to mosquitoes than longer UV wavelengths. Yellow wavelengths (570–590 nm) can be either attractive or unattractive [15, 17]. Species vary with regard to which wavelengths they are biased towards (See Additional file 1 for synthesis).

Longer wavelengths are usually less attractive to biting flies [15, 17, 18, 58, 62, 66–68, 70, 71]. However, a few studies have reported catching more Phlebotomine sand flies with red wavelengths (620–750 nm) than with lights emitting shorter wavelengths [46, 72, 73]. Sand flies are anautogenous, meaning they primarily feed on sugar and only require a blood meal to produce viable eggs. An attraction to longer wavelengths may help sand flies locate food plants [73], although this theory fails to explain why mosquitoes and midges, which are also anautogenous, do not seem to share this red attraction. In one study, resting boxes illuminated with red or infrared wavelengths caught more mosquitoes than boxes emitting shorter wavelengths [66], raising the possibility that red-attracted sand flies were seeking a resting place. Further studies are necessary to determine why high numbers of sand flies were caught using long wavelength LEDs. High catches with short wavelength light and small catches with long wavelength light are consistent with fly spectral sensitivities (Fig. 1), suggesting that spectral sensitivities can be used to predict wavelength attractiveness in some taxa.

Colour vision has not been conclusively shown in the Culicidae, Ceratopogonidae or Phlebotominae. However, a few studies have examined the attractiveness of a wavelength over a range of intensities. For *Culicoides brevitarsis*, catches were higher for the green (520 nm) wavelength than for broad-spectrum incandescent light even when the green light was half the intensity of the incandescent [18]. Catches of *Culicoides sonorensis* were higher under UV (395 nm) light than under blue (460 nm) light, despite the latter being twice as intense [70]. Similarly, *Lutzomyia longipalpis* catches were higher under UV (350 nm) and blue/green (490–546 nm) light than under a violet (400 nm) control light, regardless of whether the former were lower, equal or higher in intensity than the control [64]. Further studies are needed to confirm the existence of colour vision in these species. Wavelength discrimination independent of intensity has not yet been demonstrated in a mosquito.

### Intensity

Traps using more powerful lights tend to catch more mosquitoes, midges and sand flies than those using dimmer ones [46, 49, 71, 74–76], and increasing the intensity of a given wavelength will generally increase the attractiveness of the light [18, 76–78]. Two studies have suggested an upper threshold of intensity above which biting fly catches either reach an asymptote [18] or begin to decrease [77]—although such a threshold requires corroboration. It is likely that upper threshold varies by taxa and wavelength. Recording thresholds would ensure energy is not wasted by increasing intensity beyond that where insect catches no longer increase, as well as preventing unnecessary light pollution.

Studies into the attractiveness of specific wavelengths often do not control for light intensity. When ‘green’ and ‘blue’ LEDs are used, the ‘green’ almost always has a higher luminous intensity—the quantity of visible light emitted by a source at a given angle—than the ‘blue’ [15, 59, 60, 61, 62, 66, 69, 72, 73]. This is because LED brightness is standardised against the human eye. Two lights that appear equally bright to humans may be noticeably different to other animals. The larger catches around LEDs emitting green wavelengths may therefore be a result of the greater luminous intensity of these LEDs, and not a result of the wavelength. Complicating matters is that the attractiveness of one wavelength over another is influenced by whether both lights are at a low, medium, or high intensity. In one study, a blue (470 nm) LED attracted a higher number of *Anopheles* mosquitoes than a green (520 nm) LED of equal luminous intensity [76], but increasing the intensity of the green LED had a larger effect on *Anopheles* catches than increasing the intensity of the blue. Similarly, a blue (470 nm) LED was found to be more attractive than the equivalent green (520 nm) LED to sand flies, yet increasing luminous intensity significantly increased sand fly catches with the green LED but not the blue LED [78]. Finally, in choice-chamber experiments, when all the lights were at a low intensity, slightly more *Lutzomyia longipalpis* sand flies were attracted to blue–green (490–546 nm) wavelengths than they were the UV (350 nm) wavelength. However, when all the lights were at a higher intensity, more sand flies were attracted to UV light than the blue/green light [64]. This interaction between intensity and wavelength occurs because the different photoreceptor classes have differing sensitivities to light [64].

As users of domestic lights and conductors of vector surveys will use a variety of light intensities—due to cost, application and availability limiting the strength of the power supply—understanding the relationship between wavelength and intensity will allow for the most appropriate wavelength to be chosen for a given intensity.

### Contrast

As nocturnal insects have poor visual resolution, contrast with the background is an important component of visual attraction in host location and in flight [42, 79]. Studies on the visual attraction of biting flies have shown that the attractiveness of an object can be influenced by surrounding vegetation and ambient lighting. Green cloth was found to be less attractive than its spectral reflectivity would suggest when used against a green, spruce background [80]. Conversely, red cloth was more attractive when against that same background. In a study on the colour preferences of the mosquito *Mansonia perturbans*, white-coloured traps were unattractive during the day but highly attractive at night. The reverse was seen for the blue-coloured trap [81].

There has been little research into how environment affects the conspicuousness of emitted light (direct from a light source) as opposed to reflected light (colour of an object). Insects may behave differently towards emitted light and reflected light of the same ‘colour’. For example, red objects are reported to be attractive to mosquitoes [80–82], whereas red light is not (see section Wavelength). Understanding how the environment affects the visibility of certain lights could help to explain conflicting results for the same species [60, 61]. It could also potentially identify the most conspicuous wavelength of light for a given environment for vector surveillance and inform homeowners which colour backgrounds increase/decrease the attractiveness of white domestic lighting.

### Competing light sources

It is well established that light traps decrease in effectiveness as ambient light levels increase. When using light traps, it is sometimes recommended that sampling not take place on nights during or around a full moon due to the reduced catches on these nights [62, 83–89]. These reduced catches are unlikely to be caused by low insect flight activity. Mosquito flight activity has been shown to be higher during the full moon when trapping with truck, suction and animal-baited traps [90, 91]. Lunar phase had a significant effect on light trap catches, but not on sticky trap catches [89]. The reduced ability of the light trap to collect insects may be due to competition between light from the moon and that from the light trap [92]. Furthermore, light traps are not considered a suitable monitoring tool in northern latitudes where light levels do not fall below twilight, or in areas with significant light pollution [93–95]. High levels of background illumination may reduce the contrast and, therefore, attractiveness of the trap [96].

Vector surveillance may become more difficult as light pollution increasingly pervades rural areas where vectors are endemic [97]. However, background illumination appears to have a stronger effect on some lights more than others. Moonlight reduced catches more with incandescent light than with green (520 nm) LEDs [62]. Researchers may be able to mitigate the impact high ambient lighting, from moonlight or light pollution, has on vector surveillance by using LEDs emitting certain wavelengths.

### Range of attraction

The ‘range of attraction’ is defined as the maximum distance from which a light begins to attract insects. Knowledge of attraction ranges is used to determine how far apart light traps must be for them to not influence each other during sampling. Light traps with larger attraction ranges are able to sample insects from a wider area, which increases catch sizes and reduces the number of traps needed to sample a given area. Domestic lights with large attraction ranges would potentially attract a higher number of vectors. Attraction range is likely influenced by many factors, such as light intensity, bulb type, host presence, environment and study species [10, 98, 99].

For *Lutzomyia* sand flies, the range of attraction of an incandescent light trap has been estimated to be between 2 and 6 m [100, 101], and for *Anopheles* mosquitoes, < 5 m [102]. Studies on *Culicoides* midges, however, have produced highly variable results. The range of attraction of a Centres for Disease Control and Prevention (CDC) UV-light trap was approximately 15 m [103], and for the Onderstepoort black-light trap, the range has been estimated as ~1 m [99], ~3 m [10] or as high as ~30 m [98]. The studies with the large attraction ranges [98, 103] did not have livestock in the general vicinity of the light traps, whereas the studies with small attraction ranges [10, 99] did. Thus, additional olfactory cues may have caused the considerably shorter attraction ranges. Flies may have been primarily attracted to the animal and only responded to the light traps when at close proximity.

### Flicker

The critical fusion frequency (CFF) is the frequency at which a flickering light becomes indistinguishable from a continuous light source. Human eyes have a CFF of 50–60 Hz, whereas diurnal flying insects, including the tsetse fly, have CFF of > 100 Hz [104]. Nocturnal insects have an average CFF of 70 Hz, although the average for nocturnal, flying insects is likely to be higher due to the visual demands of flight [57]. The higher CFFs of insects imply they can perceive flickering that humans cannot, and this may affect light attraction.

Very few studies have examined fly behaviour towards flickering lights. For white fluorescent lights, the mosquito, *Culex quinquefasciatus*, and the housefly, *Musca domestica*, were found to be more attracted to the direct current (DC)-powered, non-flickering light than the alternating current (AC)-powered, flickering light [105]. In another study, fewer Diptera were also caught with white LEDs flickering at 120 Hz than with LEDs with a constant light output [57]. Finally, in two choice experiments between flickering and non-flickering white fluorescent lamps, lamps flickering at 10 and 4 Hz were less attractive to *M. domestica* than the 40,000 Hz control [106]. There was no difference in terms of attraction between the control light and light frequencies > 10 Hz. The author of the study noted that the 10 and 4 Hz lights caused flies to exhibit an “*escape response*” towards the non-flickering lamp and suggested that the sudden reduction in light intensity mimics an attack from a predator. However, in another study on UV fluorescent lights, the 100 Hz flickering light caught more *M. domestica* than a DC-powered, non-flickering light [107]. In that study, the flickering light was more attractive even at half the intensity of the non-flickering light. Whether flicker is considered attractive may be influenced by the spectral composition of the light: a flickering UV light may be attractive whereas a flickering white light may not.

The effects of flicker on biting flies require further investigation. Domestic lights traditionally operate on AC, where the current alternates on and off. LEDs react to these current changes much quicker than incandescent and fluorescent lights, resulting in a more pronounced flicker. If flickering lights are less attractive, then this is an added benefit to using LEDs for external lighting. Changes to the flicker frequencies of domestic lights could also allow for lights to be made less attractive without impoverishing the colour rendering.

## Conclusions

To improve biting fly capture rates, it is recommended to use high-intensity, short wavelength LEDs; and to trap in areas with no competing light sources. Green light has the advantage over the similarly attractive UV light, as the catches are ‘cleaner’—collecting fewer non-target insects, like Lepidoptera [19].

A minimally attractive light source would be a dim, red LED. However, the fewer the wavelengths composing a light source, the poorer the colour rendering—defined as the ability of a light to accurately represent the colours of objects [57]. With lights intended for home illumination, it is important to strike a balance between colour rendering and attractiveness to insects. It is also important to keep in mind that conditions such as colour blindness may exacerbate any colour rendering issues the light has. White light can be created by combining three or two narrow wavelengths (e.g. red–green–blue, blue–yellow, red–cyan). These lights would have improved colour rendering over a mono-chromatic light. It is unknown whether biting flies would perceive a white light composed of few, narrow wavelengths as being equally attractive as a broad-spectrum white light. However, for a light composed of few wavelengths to be equally bright as a broad-spectrum light, the intensity of those wavelengths would need to be higher. Increasing the intensity of certain wavelengths may counteract the effects of removing other wavelengths, resulting in a light that is no less attractive than a typical white light. Another potential solution is to reduce the strength of short-range wavelengths in a broad-spectrum light, thereby creating a ‘warm’ toned white light. This could potentially reduce the attractiveness of the light whilst keeping the colour rendering relatively high. However, experiments comparing insect catches between white LEDs of subtly different spectral emissions have produced conflicting results [54, 56, 108–110], and further research is therefore required.

If the spectral composition of a lamp cannot be altered, changing other aspects of the light, such as background contrast, intensity or flicker might still reduce its attractiveness. For example, homeowners may be advised which colours to paint their house to reduce the attractiveness of their outdoor lighting. Homeowners, particularly those in countries with high rates of vector-borne disease, may be willing to accept a minor reduction in light brightness. Any benefits of light flickering must also be weighed against the health risks, especially to those with photosensitive epilepsy.

 A number of studies have shown that *Lutzomyia longipalpis*, *Culicoides brevitarsis,* and *Culicoides sonorensis* are more attracted to certain wavelengths over others regardless of light intensity [18, 64, 70]. These insects may possess colour vision. A convincing test for colour vision was pioneered by von Frisch [111], who trained honeybees to associate blue or yellow cards with a sugar solution, and then had the bees choose between the coloured card and multiple shades of grey. It is assumed that if the animal is only using achromatic signals, then at least one shade of grey would be indistinguishable from the training colour. Through these experiments, von Frisch was able to prove that honeybees possess colour vision. Mosquitoes can also be trained to associate sugar solution with certain visual cues [27], suggesting similar colour vision experiments can be performed on flies.

**Fig. 3:**
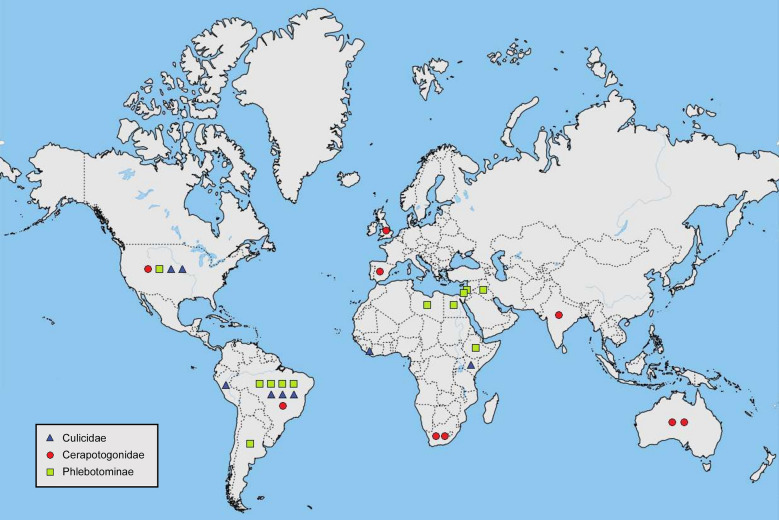
Map showing the countries where field studies into wavelength preferences on Culicidae (triangles), Ceratopogonidae (circles), and Phlebotominae (squares) have been carried out. Map is adapted from QGIS

Further studies are needed to corroborate which wavelengths are attractive (Additional file 1) and gain data on more species. A comprehensive list of species-specific differences would potentially allow for surveys to be developed for particular taxa. In this review, almost half of the studies examining the attractiveness of various wavelengths took place in the Americas (Fig. 3). Few studies have been carried out in sub-Saharan African countries, and fewer still in Asian-Pacific countries, even though these countries would greatly benefit from information on vector light attraction. Not only is the burden of vector-borne disease high in these countries [4], but the difficultly in acquiring olfactory bait means light trapping is the easiest and most reliable surveillance method [112].

Behavioural data would also benefit from supporting physiological data. The behaviour of sand flies in response to red light has led to suggestions that this family possesses long wavelength receptors [72, 73], although Mellor et al. [33] found no evidence of a long wavelength receptor; therefore, further investigation is needed. Additionally, no sensitivity data have been collected for the Ceratopogonidae. *Culicoides* species can potentially be divided into two groups in terms of light preference, namely green-attracted and UV-attracted [19], and the spectral sensitivities may reflect this grouping. Physiological studies on a wider variety of species may reveal differences between families or between diurnal and nocturnal flies.

Understanding how wavelength, intensity, background contrast, range of attraction and light flicker interact with each other would provide a more complete picture of light attraction in biting flies. More research is also needed on aspects of light attraction not discussed in this review, such as light height [113, 114], light polarisation [115], time of day effects [40] and the presence of reflective surfaces [72]. Finally, future studies should control for thermal emissions, due to the attractiveness of IR light, as well as intensity, as its effects may not be consistent across wavelengths [64, 76, 78].

In summary, in this review we outline how light trapping can be made more efficient and we highlight how, despite current knowledge of how to reduce insect attraction to lights, modifying domestic lights remains as a challenging though potentially important research direction.

## Supplementary Information

**Additional file 1**. Wavelength preferences/biases of various species of Ceratopogonidae, Culicidae, and Phlebotominae in experiments comparing catches between lights of different wavelengths. What constitutes a preference/bias was determined on a case-by-case basis. Species with inconclusive preferences/biases are not listed here. Colour is used in cases where dominant wavelength is not specified.

## Data Availability

The data generated during this study is included in this published article in Additional file 1.

## Abbreviations

CDC: Centres for Disease Control
CFL: Compact fluorescent lamp
CFF: Critical fusion frequency
IR: Infrared
LED: Light-emitting diode
R: Retinula cell
Rh: Rhodopsin
UV: Ultraviolet

## References

[1] Lehane MJ (2005). The biology of blood-sucking in insects.

[2] World Health Organization (1996). The world health report: 1996: fighting disease, fostering development.

[3] Mellor PS, Boorman J, Baylis M (2000). *Culicoides* biting midges: their role as arbovirus vectors. Annu Rev Entomol.

[4] World Health Organization (2004). The world health report: 2004: changing history.

[5] Hayes RO, Bellamy RE, Reeves WC, Willis MJ (1958). Comparison of four sampling methods for measurement of *Culex tarsalis* adult populations. Mosquito News..

[6] Service MW (1977). Critical review of procedures for sampling populations of adult mosquitos. Bull Entomol Res.

[7] Service MW (1993). Sampling the adult resting population. Mosquito ecology: field sampling methods.

[8] Mboera LEG (2005). Sampling techniques for adult Afrotropical malaria vectors and their reliability in the estimation of entomological inoculation rate. Tanzania J Health Res.

[9] Tchouassi DP, Sang R, Sole CL, Bastos AD, Cohnstaedt LW, Torto B (2012). Trapping of Rift Valley Fever (RVF) vectors using Light Emitting Diode (LED) CDC traps in two arboviral disease hot spots in Kenya. Parasites Vectors.

[10] Venter GJ, Majatladi DM, Labuschagne K, Boikanyo SN, Morey L (2012). The attraction range of the Onderstepoort 220 V light trap for *Culicoides* biting midges as determined under South African field conditions. Vet Parasitol.

[11] Faiman R, Cuño R, Warburg A (2009). Comparative efficacy of three suction traps for collecting phlebotomine sand flies (Diptera: Psychodidae) in open habitats. J Vector Ecol.

[12] Sudia WD, Chamberlain RW (1962). Battery-operated light trap, an improved model. Mosquito News.

[13] Service MW (1970). A battery-operated light-trap for sampling mosquito populations. Bull World Health Organ.

[14] Addison LD, Watson BG, Webber LA (1979). Apparatus for the use of CO_2_ gas with a CDC light trap. Mosquito News.

[15] Burkett DA, Butler JF, Kline DL (1998). Field evaluation of colored light-emitting diodes as attractants for woodland mosquitoes and other Diptera in north central Florida. J Am Mosq Control Assoc.

[16] Cohnstaedt L, Gillen JI, Munstermann LE (2008). Light-emitting diode technology improves insect trapping. J Am Mosq Control Assoc.

[17] Rogers E, Sholdt LL, Falcón R (1993). Effects of incorporating chemical light sources in CDC traps: differences in the capture rates of neotropical *Culex*, *Anopheles* and *Uranotaenia* (Diptera: Culicidae). Pan Pac Entomol.

[18] Bishop AL, Worrall R, Spohr LJ, McKenzie HJ, Barchia IM (2004). Response of *Culicoides* spp. (Diptera: Ceratopogonidae) to light-emitting diodes. Aust J Entomol.

[19] Bishop AL, Bellis GA, McKenzie HJ, Spohr LJ, Worrall RJ, Harris AM (2006). Light trapping of biting midges *Culicoides* spp. (Diptera: Ceratopogonidae) with green light-emitting diodes. Aust J Entomol.

[20] Barghini A, de Medeiros BA (2010). Artificial lighting as a vector attractant and cause of disease diffusion. Environ Health Perspect.

[21] Pacheco-Tucuch FS, Ramirez-Sierra MJ, Gourbière S, Dumonteil E (2012). Public street lights increase house infestation by the Chagas disease vector *Triatoma dimidiata*. PLoS ONE.

[22] Dumonteil E, Nouvellet P, Rosecrans K, Ramirez-Sierra MJ, Gamboa-León R, CruzChan V (2013). Eco-bio-social determinants for house infestation by non-domiciliated *Triatoma dimidiata* in the Yucatan Peninsula, Mexico. PLoS Negl Trop Dis.

[23] Taylor B (1997). Malaria transmission—mosquitoes, humans and their behaviour. Antenna..

[24] Yamamoto S, Louis VR, Sie A, Sauerborn R (2010). Household risk factors for clinical malaria in a semi-urban area of Burkina Faso: a case–control study. Trans R Soc Trop Med Hyg.

[25] Pellegrini L, Tasciotti L (2016). The electrification–malaria nexus: the case of rural Uganda. Eur J Dev Res.

[26] Tasciotti L (2017). Use of electricity and malaria occurrence: Is there a link? The case of Malawi. Energy Policy.

[27] Bernáth B, Anstett V, Guerin PM (2016). *Anopheles gambiae* females readily learn to associate complex visual cues with the quality of sugar sources. J Insect Physiol.

[28] Warrant E. Invertebrate vision. In: Roitberg BD, editor. Reference module in life sciences. Elsevier. Amsterdam: Elsevier; 2017. p. 1–14.

[29] Gao S, Takemura SY, Ting CY, Huang S, Lu Z, Luan H (2008). The neural substrate of spectral preference in *Drosophila*. Neuron.

[30] Briscoe AD, Chittka L (2001). The evolution of color vision in insects. Annu Rev Entomol.

[31] Muir LE, Thorne MJ, Kay BH (1992). *Aedes aegypti* (Diptera: Culicidae) vision: spectral sensitivity and other perceptual parameters of the female eye. J Med Entomol.

[32] Peach DA, Ko E, Blake AJ, Gries G (2019). Ultraviolet inflorescence cues enhance attractiveness of inflorescence odour to *Culex pipiens* mosquitoes. PLoS ONE.

[33] Mellor HE, Hamilton JG, Anderson M (1996). Spectral sensitivity in the eyes of male and female *Lutzomyia longipalpis* sandflies. Med Vet Entomol.

[34] Hardie R, Vogt K, Rudolph A (1989). The compound eye of the tsetse fly (*Glossina morsitans morsitans* and *Glossina palpalis palpalis*). J Insect Physiol.

[35] Allan SA, Stoffolano JG, Bennett RR (1991). Spectral sensitivity of the horse fly *Tabanus nigrovittatus* (Diptera: Tabanidae). Can J Zool.

[36] Kirschfeld K, Vogt K (1986). Does retinol serve a sensitizing function in insect photoreceptors?. Vision Res.

[37] Agee HR, Patterson RS (1983). Spectral sensitivity of stable, face, and horn flies and behavioral responses of stable flies to visual traps (Diptera: Muscidae). Environ Entomol.

[38] Yamaguchi S, Desplan C, Heisenberg M (2010). Contribution of photoreceptor subtypes to spectral wavelength preference in *Drosophila*. Proc Natl Acad Sci USA.

[39] Baik LS, Recinos Y, Chevez JA, Holmes TC (2018). Circadian modulation of light-evoked avoidance/attraction behavior in *Drosophila*. PLoS ONE.

[40] Baik LS, Nave C, Au DD, Guda T, Chevez JA, Ray A (2020). Circadian regulation of light-evoked attraction and avoidance behaviors in daytime-versus nighttime-biting mosquitoes. Curr Biol.

[41] Kelber A, Vorobyev M, Osorio D (2003). Animal colour vision–behavioural tests and physiological concepts. Biol Rev.

[42] Allan SA, Day JF, Edman JD (1987). Visual ecology of biting flies. Annu Rev Entomol.

[43] Agi E, Langen M, Altschuler SJ, Wu LF, Zimmermann T, Hiesinger PR (2014). The evolution and development of neural superposition. J Neurogenet.

[44] Ecodesign Regulatory Committee. Member States approve the phasing-out of incandescent bulbs by 2012. 2008. https://ec.europa.eu/commission/presscorner/detail/en/IP_08_1909. Accessed 14 June 2020.

[45] Zissis G, Bertoldi P (2018). Status of LED-lighting world market in 2017.

[46] Burkett DA, Knight R, Dennett JA, Sherwood V, Rowton E, Coleman RE (2007). Impact of phlebotomine sand flies on US military operations at Tallil Air Base, Iraq: 3. Evaluation of surveillance devices for the collection of adult sand flies. J Med Entomol..

[47] Venter GJ, Labuschagne K, Hermanides KG, Boikanyo SNB, Majatladi DM, Morey L (2009). Comparison of the efficiency of five suction light traps under field conditions in South Africa for the collection of *Culicoides* species. Vet Parasitol.

[48] Kline DL, Hogsette JA, Müller GC (2011). Comparison of various configurations of CDC-type traps for the collection of *Phlebotomus papatasi* Scopoli in southern Israel. J Vector Ecol.

[49] Obenauer PJ, Abdel-Dayem MS, Stoops CA, Villinski JT, Tageldin R, Fahmy NT (2013). Field responses of *Anopheles gambiae* complex (Diptera: Culicidae) in Liberia using yeast-generated carbon dioxide and synthetic lure-baited light traps. J Med Entomol.

[50] Li CX, Smith ML, Fulcher A, Kaufman PE, Zhao TY, Xue RD (2015). Field evaluation of three new mosquito light traps against two standard light traps to collect mosquitoes (Diptera: Culicidae) and non-target insects in northeast Florida. Florida Entomol.

[51] Müller GC, Hogsette JA, Kline DL, Beier JC, Revay EE, Xue RD (2015). Response of the sand fly *Phlebotomus papatasi* to visual, physical and chemical attraction features in the field. Acta Trop.

[52] Peck GW, Castro-Llanos F, López-Sifuentes VM, Vásquez GM, Lindroth E (2018). Comparative analysis of mosquito trap counts in the Peruvian Amazon: effect of trap type and other covariates on counts and diversity. J Am Mosq Control Assoc.

[53] van Grunsven RH, Donners M, Boekee K, Tichelaar I, van Geffen KG, Groenendijk D (2014). Spectral composition of light sources and insect phototaxis, with an evaluation of existing spectral response models. J Insect Conserv.

[54] Longcore T, Aldern HL, Eggers JF, Flores S, Franco L, Hirshfield-Yamanishi E (2015). Tuning the white light spectrum of light emitting diode lamps to reduce attraction of nocturnal arthropods. Philos Trans R Soc B Biol Sci.

[55] Justice MJ, Justice TC (2016). Attraction of insects to incandescent, compact fluorescent, halogen, and LED lamps in a light trap: implications for light pollution and urban ecologies. Entomol News.

[56] Wakefield A, Broyles M, Stone EL, Jones G, Harris S (2016). Experimentally comparing the attractiveness of domestic lights to insects: do LEDs attract fewer insects than conventional light types?. Ecol Evol.

[57] Barroso A, Haifig I, Janei V, da Silva I, Dietrich C, Costa-Leonardo AM (2017). Effects of flickering light on the attraction of nocturnal insects. Light Res Technol.

[58] Silva JS, Souto Couri M, de Leão Giupponi AP, Alencar J (2014). Mosquito fauna of the Guapiaçu Ecological Reserve, Cachoeiras de Macacu, Rio de Janeiro, Brazil, collected under the influence of different color CDC light traps. J Vector Ecol.

[59] Silva FS, Brito JM, Costa-Neta BM (2015). Field evaluation of light-emitting diode as attractant for blood-sucking midges of the genus Culicoides Latreille (Diptera: Culicomorpha, Ceratopogonidae) in the Brazilian savanna. Entomol News.

[60] Silva FS, Brito JM, Costa Neta BM, Lobo SEPD (2015). Evaluation of light emitting diodes as attractant for sandflies (Diptera: Psychodidae: Phlebotominae) in northeastern Brazil. Mem Inst Oswaldo Cruz.

[61] Silva FS, da Silva AA, Rebêlo JMM (2016). An evaluation of light-emitting diode (LED) traps at capturing phlebotomine sand flies (Diptera: Psychodidae) in a livestock area in Brazil. J Med Entomol.

[62] Costa-Neta BM, da Silva AA, Brito JM, Moraes JLP, Rebêlo JMM, Silva FS (2017). Light-emitting diode (LED) traps improve the light-trapping of anopheline mosquitoes. J Med Entomol.

[63] da Silva AA, Rebêlo JMM, Carneiro BF, Castro MPP, de Sousa de Almeida M, Ponte IS (2019). Exploiting the synergistic effect of kairomones and light-emitting diodes on the attraction of phlebotomine sand flies to light traps in Brazil. J Med Entomol.

[64] Mellor HE, Hamilton JGC (2003). Navigation of *Lutzomyia longipalpis* (Diptera: Psychodidae) under dusk or starlight conditions. Bull Entomol Res.

[65] Bishop AL, McKenzie HJ, Spohr LJ (2008). Attraction of *Culicoides brevitarsis* Kieffer (Diptera: Ceratopogonidae) and *Culex annulirostris* Skuse (Diptera: Culicidae) to simulated visual and chemical stimuli from cattle. Aust J Entomol.

[66] Bentley MT, Kaufman PE, Kline DL, Hogsette JA (2009). Response of adult mosquitoes to light-emitting diodes placed in resting boxes and in the field. J Am Mosq Control Assoc.

[67] Hope A, Gubbins S, Sanders C, Denison E, Barber J, Stubbins F (2015). A comparison of commercial light-emitting diode baited suction traps for surveillance of *Culicoides* in northern Europe. Parasites Vectors.

[68] González M, Alarcón-Elbal PM, Valle-Mora J, Goldarazena A (2016). Comparison of different light sources for trapping *Culicoides* biting midges, mosquitoes and other dipterans. Vet Parasitol.

[69] Serrano AK, Malo EA, Mikery OF, Castillo A (2016). Response of *Lutzomyia cruciata* (1) to CDC light traps modified with light-emitting diodes. Southwestern Entomol.

[70] Snyder D, Cernicchiaro N, Cohnstaedt LW (2016). Sugar-feeding status alters biting midge photoattraction. Med Vet Entomol.

[71] Venter GJ, Boikanyo SN, De Beer CJ (2018). The efficiency of light-emitting diode suction traps for the collection of South African livestock associated *Culicoides* species. Med Vet Entomol.

[72] Hoel DF, Butler JF, Fawaz EY, Watany N, El-Hossary SS, Villinski J (2007). Response of phlebotomine sand flies to light-emitting diode-modified light traps in southern Egypt. J Vector Ecol.

[73] Mann RS, Kaufman PE, Butler JF (2009). Lutzomyia spp (Diptera: Psychodidae) response to olfactory attractant-and light emitting diode-modified Mosquito Magnet X (MM-X) traps. J Med Entomol.

[74] Brazil RP (2003). Attraction of sand flies (Diptera: Psychodidae) to light traps in rural areas of Minas Gerais State, Brazil. J Am Mosq Control Assoc.

[75] Kim HC, Kim MS, Choi KS, Hwang DU, Johnson JL, Klein TA (2017). Comparison of adult mosquito black-light and light-emitting diode traps at three cowsheds located in malaria-endemic areas of the Republic of Korea. J Med Entomol.

[76] Costa-Neta BM, Lima-Neto AR, da Silva AA, Brito JM, Aguiar JVC, Ponte IS (2018). Centers for Disease Control-type light traps equipped with high-intensity light-emitting diodes as light sources for monitoring *Anopheles* mosquitoes. Acta Trop.

[77] Teodoro U, Lonardoni MVC, Silveira TGV, Dias ADC, Abbas M, Alberton D (2007). Light and hens as attraction factors of *Nyssomyia whitmani* in a rural area, Southern Brazil. Rev Saúde Pública..

[78] Lima-Neto AR, Costa-Neta BM, da Silva AA, Brito JM, Aguiar JV, Ponte IS (2018). The effect of luminous intensity on the attraction of phlebotomine sand flies to light traps. J Med Entomol.

[79] Kennedy JS (1940). The visual responses of flying mosquitoes. J Zool.

[80] Brown AWA (1954). Studies on the responses of the female Aedes mosquito. Part VI. The attractiveness of coloured cloths to Canadian species. Bull Entomol Res..

[81] Browne SM, Bennett GF (1981). Response of mosquitoes (Diptera: Culicidae) to visual stimuli. J Med Entomol.

[82] Gjullin CM (1947). Effect of clothing color on the rate of attack of *Aedes* mosquitoes. J Econ Entomol.

[83] Rubio-Palis Y (1992). Influence of moonlight on light trap catches of the malaria vector *Anopheles nuneztovari* in Venezuela. J Am Mosq Control Assoc.

[84] Janousek TE, Olson JK (1994). Effect of a lunar eclipse on the flight activity of mosquitoes in the upper Gulf Coast of Texas. J Am Mosq Control Assoc.

[85] Bishop AL, Kirkland PD, McKenzie HJ, Spohr LJ, Barchia IM, Muller MJ (1995). Distribution and seasonal movements of Culicoides brevitarsis Kieffer (Diptera: Ceratopogonidae) at the southern limits of its distribution in New South Wales and their correlation with arboviruses affecting livestock. Aust J Entomol.

[86] Mishra NSA, Curtis CF, Sharma VP (1996). Influence of moonlight on light-trap catches of the malaria vector *Anopheles culicifacies* (Diptera: Culicidae) in central India. Bull Entomol Res.

[87] Bishop AL, McKenzie HJ, Barchia IM, Spohr LJ (2000). Moon phase and other factors affecting light-trap catches of *Culicoides brevitarsis* Kieffer (Diptera: Ceratopogonidae). Aust J Entomol.

[88] Nowinszky L (2004). Nocturnal illumination and night flying insects. Appl Ecol Environ Res.

[89] Gebresilassie A, Yared S, Aklilu E, Kirstein OD, Moncaz A, Tekie H (2015). The influence of moonlight and lunar periodicity on the efficacy of CDC light trap in sampling *Phlebotomus* (*Larroussius*) *orientalis* Parrot, 1936 and other *Phlebotomus* sandflies (Diptera: Psychodidae) in Ethiopia. Parasites Vectors.

[90] Bidlingmayer WL (1964). The effect of moonlight on the flight activity of mosquitoes. Ecology.

[91] Bidlingmayer WL (1974). The influence of environmental factors and physiological stage on flight patterns of mosquitoes taken in the vehicle aspirator and truck, suction, bait and New Jersey light traps. J Med Entomol.

[92] Bowden J (1973). The influence of moonlight on catches of insects in light-traps in Africa. Part I. The moon and moonlight. Bull Entomol Res..

[93] Southwood TRE, Henderson PA (2000). Ecological methods.

[94] Meiswinkel R, Elbers ARW (2016). The dying of the light: crepuscular activity in *Culicoides* and impact on light trap efficacy at temperate latitudes. Med Vet Entomol.

[95] Medlock J, Balenghien T, Alten B, Versteirt V, Schaffner F (2018). Field sampling methods for mosquitoes, sandflies, biting midges and ticks: VectorNet project 2014–2018. EFSA Support Publ.

[96] Wilton DP (1975). Evaluations of three types of light traps for collection of *Anopheles AlblManus* Wiedeman (Diptera: Culicidae). J Med Entomol.

[97] Gaston KJ, Davies TW, Bennie J, Hopkins J (2012). Reducing the ecological consequences of night-time light pollution: options and developments. J Appl Ecol.

[98] Rigot T, Gilbert M (2012). Quantifying the spatial dependence of *Culicoides* midge samples collected by Onderstepoort-type blacklight traps: an experimental approach to infer the range of attraction of light traps. Med Vet Entomol.

[99] Elbers ARW, Meiswinkel R (2016). Limited attractant range of the black-light suction trap for the capture of *Culicoides* biting midges (Diptera: Ceratopogonidae). J Appl Entomol.

[100] Valenta DT, Tang Y, Anez N. A new method to determine the distance at which phlebotomine sandflies are attracted to light under field conditions. Proc 2nd Int Symp on Phlebotomine Sand Flies (ISOPS II), Merida, Venezuela. Bol Direccion Malar Saneamiento Ambiental. 1995;35:353–8.

[101] Campbell-Lendrum D, Pinto MC, Davies C (1999). Is *Lutzomyia intermedia* (Lutz and Neiva, 1912) more endophagic than *Lutzomyia whitmani* (Antunes and Coutinho, 1939) because it is more attracted to light?. Memó Inst Oswaldo Cruz..

[102] Costantini C, Sagnon NF, Sanogo E, Merzagora L, Coluzzi M (1998). Relationship to human biting collections and influence of light and bednet in CDC light-trap catches of West African malaria vectors. Bull Entomol Res.

[103] Kirkeby C, Græsbøll K, Stockmarr A, Christiansen LE, Bødker R (2013). The range of attraction for light traps catching *Culicoides* biting midges (Diptera: Ceratopogonidae). Parasites Vectors.

[104] Inger R, Bennie J, Davies TW, Gaston KJ (2014). Potential biological and ecological effects of flickering artificial light. PLoS ONE.

[105] Chu C, Chen TY, Henneberry TJ (2006). Attractiveness of flickering and nonflickering cool white fluorescent light to *Culex quinquefasciatus*, *Musca domestica* and *Pectinophora gossypiela* adults, and *Acheta domesticus* and *Periplaneta americana* nymphs. Southwest Entomol.

[106] Smallegange RC. Attractiveness of different light wavelengths, flicker frequencies and odours to the housefly (*Musca domestica* L.). PhD thesis. Groningen: Rijksuniversiteit Groningen; 2003.

[107] Syms PR, Goodman LJ (1987). The effect of flickering U-V light output on the attractiveness of an insect electrocutor trap to the house-fly, Musca domestica. Entomol Exp Appl.

[108] Eisenbeis G, Eick K (2011). Studie zur Anziehung nachtaktiver Insekten an die Straßenbeleuchtung unter Einbeziehung von LEDs. Natur Landschaft.

[109] Pawson SM, Bader MF (2014). LED lighting increases the ecological impact of light pollution irrespective of color temperature. Ecol Appl.

[110] Spoelstra K, van Grunsven RH, Donners M, Gienapp P, Huigens ME, Slaterus R (2015). Experimental illumination of natural habitat—an experimental set-up to assess the direct and indirect ecological consequences of artificial light of different spectral composition. Phil Trans R Soc B Biol Sci.

[111] von Frisch K (1914). Der farbensinn und formensinn der biene.

[112] Smallegange RC, Schmied WH, van Roey KJ, Verhulst NO, Spitzen J, Mukabana WR (2010). Sugar-fermenting yeast as an organic source of carbon dioxide to attract the malaria mosquito Anopheles gambiae. Malaria J.

[113] Braverman Y, Linley JR (1993). Effect of light trap height on catch of *Culicoides* (Diptera: Ceratopogonidae) in Israel. J Med Entomol.

[114] Shone SM, Glass GE, Norris D (2006). Targeted trapping of mosquito vectors in the Chesapeake Bay Area of Maryland. J Med Entomol.

[115] Bernáth B, Horváth G, Gál J, Fekete G, Meyer-Rochow VB (2008). Polarized light and oviposition site selection in the yellow fever mosquito: no evidence for positive polarotaxis in *Aedes aegypti*. Vision Res.

[116] Olanrewaju HA, Miller WW, Maslin WR, Collier SD, Purswell JL, Branton SL (2016). Effects of light sources and intensity on broilers grown to heavy weights. Part 1: growth performance, carcass characteristics, and welfare indices. Poult Sci.

