# Supplementary material for: Artificial light and biting flies: the parallel development of attractive light traps and unattractive domestic lights
Source: Parasit Vectors. 2021 Jan 7;14:28. doi: 10.1186/s13071-020-04530-3 (PMC7789162; doi:10.1186/s13071-020-04530-3)
Supplement: Supplementary file 1 — Additional file 1. Wavelength preferences/biases of various species of Ceratopogonidae, Culicidae, and Phlebotominae in experiments comparing catches between lights of different wavelengths. What constitutes a preference/bias was determined on a case-by-case basis. Species with inconclusive preferences/biases are not listed here. Colour is used in cases where dominant wavelength is not specified. [file 13071_2020_4530_MOESM1_ESM.docx]

**ADDITIONAL FILE 1**

| **Family** | **Species** | **Attractive wavelengths** | **Unattractive wavelengths** | **Location** | **Reference** | **Lights compared** |
| --- | --- | --- | --- | --- | --- | --- |
| Ceratopogonidae | *Culicoides actoni* (Smith) | 520 nm |  | New South Wales, Australia | [19] | Incandescent and LEDs (UV, blue, green, yellow) |
|  | *Culicoides anophelis* (Edwards) | 570 nm |  | India | [119] | LEDs (UV, green) |
|  | *Culicoides austropalpalis* (Lee & Reye) | 475 nm | 640 nm | New South Wales, Australia | [18] | Incandescent and LEDs (white, blue, green, yellow, red) |
|  |  | UV | Incandescent & 595 nm | New South Wales, Australia | [19] | Incandescent and LEDs (UV, blue, green, yellow) |
|  | *Culicoides bedfordi* (Ingram & Macfie) | UV |  | South Africa | [122] | Incandescent and UV |
|  | *Culicoides bolitinos* (Meiswinkel) | UV |  | South Africa | [122] | Incandescent and UV |
|  | *Culicoides brevipalpis* (Delfinado) | 520 nm |  | New South Wales, Australia | [19] | Incandescent and LEDs (UV, blue, green, yellow) |
|  | *Culicoides brevitarsis* (Kieffer) | 520 nm | 640 nm | New South Wales, Australia | [18] | Incandescent and LEDs (white, blue, green, yellow, red) |
|  |  | 520 nm | 595 nm | New South Wales, Australia | [19] | Incandescent and LEDs (UV, blue, green, yellow) |
|  |  | 390 nm |  | India | [119] | LEDs (UV, green) |
|  | *Culicoides brunnicans* (Edwards) | 570 nm | 660 nm | United Kingdom | [67] | UV and LEDs (white, UV, blue, green, yellow, red) |
|  | *Culicoides bundyensis* (Lee & Reye) | 520 nm | 640 nm | New South Wales, Australia | [18] | Incandescent and LEDs (white, blue, green, yellow, red) |
|  |  | 520 nm |  | New South Wales, Australia | [19] | Incandescent and LEDs (UV, blue, green, yellow) |
|  | *Culicoides bunrooiensis* (Lee & Reye) | 475 nm |  | New South Wales, Australia | [18] | Incandescent and LEDs (white, blue, green, yellow, red) |
|  |  | UV | Incandescent & 595 nm | New South Wales, Australia | [19] | Incandescent and LEDs (UV, blue, green, yellow) |
|  | *Culicoides cataneii* (Clastrier) | UV | 660 nm & white LED | Spain | [68] | LEDs (white, UV, blue, green, red) |
|  | *Culicoides dewulfi* (Goetghebuer) | 570 nm | 660 nm | United Kingdom | [67] | UV and LEDs (white, UV, blue, green, yellow, red) |
|  | *Culicoides duartei* (Tavares & Luna Dias) | 520 nm |  | Maranhão, Brazil | [59] | Incandescent and LEDs (blue, green) |
|  | *Culicoides dycei* (Lee & Reye) | 475 nm | 640 nm | New South Wales, Australia | [18] | Incandescent and LEDs (white, blue, green, yellow, red) |
|  |  | UV | Incandescent & 595 nm | New South Wales, Australia | [19] | Incandescent and LEDs (UV, blue, green, yellow) |
|  | *Culicoides enderleini* (Cornet & Brunhes) | UV |  | South Africa | [122] | Incandescent and UV |
|  |  | UV | Red | South Africa | [71] | UV and LEDs (white, blue, green, red) |
|  | Culicoides exspectator (Clastrier) | UV |  | South Africa | [122] | Incandescent and UV |
|  | *Culicoides fulvus* (Sen & Das Gupta) | 520 nm |  | New South Wales, Australia | [19] | Incandescent and LEDs (UV, blue, green, yellow) |
|  | *Culicoides imicola* (Kieffer) | UV |  | South Africa | [122] | Incandescent and UV |
|  |  | UV | Red | South Africa | [71] | UV and LEDs (white, blue, green, red) |
|  |  | 390 nm |  | India | [119] | LEDs (UV, green) |
|  | *Culicoides impunctatus* (Goetghebuer) |  | 660 nm | United Kingdom | [67] | UV and LEDs (white, UV, blue, green, yellow, red) |
|  | *Culicoides leucostictus* (Kieffer) | UV |  | South Africa | [122] | Incandescent and UV |
|  | *Culicoides lutzi* (Costa Lima) | 520 nm |  | Maranhão, Brazil | [59] | Incandescent and LEDs (blue, green) |
|  | *Culicoides magnus* (Colaco) | UV |  | South Africa | [122] | Incandescent and UV |
|  | *Culicoides marksi* (Lee & Reye) | 475 nm | 640 nm | New South Wales, Australia | [18] | Incandescent and LEDs (white, blue, green, yellow, red) |
|  |  | UV | 595 nm | New South Wales, Australia | [19] | Incandescent and LEDs (UV, blue, green, yellow) |
|  | *Culicoides narrabeenensis* complex | 520 nm |  | New South Wales, Australia | [19] | Incandescent and LEDs (UV, blue, green, yellow) |
|  | *Culicoides nattiensis complex* | 475 nm | 640 nm, 595 nm & white LED | New South Wales, Australia | [18] | Incandescent and LEDs (white, blue, green, yellow, red) |
|  |  | 520 nm | Incandescent & 595 nm | New South Wales, Australia | [19] | Incandescent and LEDs (UV, blue, green, yellow) |
|  | Culicoides nivosus (de Meillon) | UV |  | South Africa | [122] | Incandescent and UV |
|  | *Culicoides obsoletus* (Meigen) | UV | 660 nm | United Kingdom | [67] | UV and LEDs (white, UV, blue, green, yellow, red) |
|  |  |  | 660 nm | Spain | [68] | LEDs (white, UV, blue, green, red) |
|  | *Culicoides ornatus* (Taylor) | 520 nm |  | New South Wales, Australia | [19] | Incandescent and LEDs (UV, blue, green, yellow) |
|  | *Culicoides oxystoma* (Kieffer) | 520 nm |  | New South Wales, Australia | [19] | Incandescent and LEDs (UV, blue, green, yellow) |
|  |  | 390 nm |  | India | [119] | LEDs (UV, green) |
|  | *Culicoides pallidothorax* (Lee & Reye) | 520 nm |  | New South Wales, Australia | [19] | Incandescent and LEDs (UV, blue, green, yellow) |
|  | *Culicoides paucienfuscatus* (Barbosa) | Incandescent | 470 nm | Maranhão, Brazil | [59] | Incandescent and LEDs (blue, green) |
|  | *Culicoides peregrinus* (Macfie) | 520 nm |  | New South Wales, Australia | [19] | Incandescent and LEDs (UV, blue, green, yellow) |
|  |  | 390 nm |  | India | [119] | LEDs (UV, green) |
|  | *Culicoides pulicaris* (Linnaeus) | UV | 660 nm | United Kingdom | [67] | UV and LEDs (white, UV, blue, green, yellow, red) |
|  | *Culicoides punctatus* (Meigen) | UV | 660 nm | United Kingdom | [67] | UV and LEDs (white, UV, blue, green, yellow, red) |
|  |  | UV | 660 nm | Spain | [68] | LEDs (white, UV, blue, green, red) |
|  | *Culicoides pycnostictus* (Ingram & Macfie) | UV |  | South Africa | [122] | Incandescent and UV |
|  | *Culicoides reevesi* (Wirth) |  | Incandescent & UV | California & Utah, USA | [118] | Incandescent and UV |
|  | *Culicoides scoticus* (Downes & Kettle) | UV | 660 nm | United Kingdom | [67] | UV and LEDs (white, UV, blue, green, yellow, red) |
|  | Culicoides similis (Carter) | UV |  | South Africa | [122] | Incandescent and UV |
|  | *Culicoides sonorensis* (Wirth & Jones) | 355 nm & 365 nm | 640 nm & 560 nm | Lab | [70] | LEDs (UV, blue, green, red) |
|  | *Culicoides victoriae* (Macfie) | 520 nm | 640 nm | New South Wales, Australia | [18] | Incandescent and LEDs (white, blue, green, yellow, red) |
|  |  | 520 nm | Incandescent & UV | New South Wales, Australia | [19] | Incandescent and LEDs (UV, blue, green, yellow) |
|  | *Culicoides wadai* (Kitaoka) | 520 nm |  | New South Wales, Australia | [19] | Incandescent and LEDs: UV, blue, green, yellow |
|  | *Culicoides zuluensis* (de Meillon) | UV |  | South Africa | [122] | Incandescent and UV |
| Culicidae | *Aedes aegypti* (Linnaeus) | 365 nm (night-time) |  | Lab | [40] | LEDs (UV, blue, red) |
|  | *Aedes dupreei* (Coquillett) |  | 587 nm | Florida, USA | [15] | Incandescent and LEDs (blue, green, yellow, orange, red, IR) |
|  | *Aedes infimatus* (Dyar & Knab) | 470 nm | 660 nm & 860 nm | Florida, USA | [66]* | LEDs (blue, green, red, IR) |
|  | *Aedes tricholabis*(Edwards) | Incandescent | 660 nm | Kenya | [9] | Incandescent and LEDs (BGR, violet, blue, green, red) |
|  | *Aedes vexans* (Meigen) | 470 nm | 660 nm & 860 nm | Florida, USA | [66]* | LEDs (blue, green, red, IR) |
|  | *Aediomyia squamipennis* (Lynch) |  | Red chemical stick | Peru | [17] | Incandescent and chemical sticks (white, blue, green, yellow, red) |
|  | *Anopheles coluzzii* (Coetzee & Wilkerson) | 630 nm (day-time)  365 nm (night-time) | 365 nm (day-time)  630 nm (night-time) | Lab | [40] | LEDs (UV, blue, red) |
|  | *Anopheles crucians* (Wiedemann) | Incandescent | 940 nm | Florida, USA | [15] | Incandescent and LEDs (blue, green, yellow, orange, red, IR) |
|  | *Anopheles gambiae s.s*(Giles) | Fluorescent UV |  | Liberia | [49] | Incandescent, fluorescent UV, and LED UV |
|  | *Anopheles argyritarsis* (Robineau) | 520 nm | Incandescent | Maranhão, Brazil | [62] | Incandescent and LEDs (blue, green) |
|  |  | 470 nm |  | Maranhão, Brazil | [76] | LEDs (blue, green) |
|  | *Anopheles evansae* (Brethés) | 520 nm | Incandescent | Maranhão, Brazil | [62] | Incandescent and LEDs (blue, green) |
|  | *Anopheles goeldii* (Rozeboom & Gabaldón) | 470 nm & 520 nm | Incandescent | Maranhão, Brazil | [62] | Incandescent and LEDs (blue, green) |
|  |  | 470 nm |  | Maranhão, Brazil | [76] | LEDs (blue, green) |
|  | *Anopheles mattogrossensis* (Lutz & Neiva) | Yellow chemical stick |  | Peru | [17] | Incandescent and chemical sticks (white, blue, green, yellow, red) |
|  | *Anopheles triannulatus* s.l. (Neiva & Pinto) | 520 nm | Incandescent | Maranhão, Brazil | [62] | Incandescent and LEDs (blue, green) |
|  | *Coquillettidia fasciolata* (Lynch) | 570 nm |  | Maranhão, Brazil | [58] | Incandescent and LEDs (UV, blue, green, red) |
|  | *Coquillettidia juxtamansonia* (Chagas) | 570 nm | 660 nm | Maranhão, Brazil | [58] | Incandescent and LEDs (UV, blue, green, red) |
|  | *Coquillettidia perturbans* (Walker) | 502 nm | 860 nm | Florida, USA | [66]* | LEDs (blue, green, red, IR) |
|  | *Culex declarator* (Dyar & Knab) | Incandescent | Red chemical stick | Peru | [17] | Incandescent and chemical sticks (white, blue, green, yellow, red) |
|  |  | 430 nm | 660 nm | Maranhão, Brazil | [58] | Incandescent and LEDs (UV, blue, green, red) |
|  | *Culex adamesi* (Sirivanakam) |  | Red chemical stick | Peru | [17] | Incandescent and chemical sticks (white, blue, green, yellow, red) |
|  | *Culex amazonensis* (Lutz) | White chemical stick | Red chemical stick | Peru | [17] | Incandescent and chemical sticks (white, blue, green, yellow, red) |
|  | *Culex erraticus* (Dyar and Knab) | 470 nm | 860 nm | Florida, USA | [66]* | LEDs (blue, green, red, IR) |
|  | *Culex nigripalpus* (Theobald) | 470 nm | 660 nm & 860 nm | Florida, USA | [66]* | LEDs (blue, green, red, IR) |
|  |  | 430 nm | Incandescent & 660 nm | Maranhão, Brazil | [58] | Incandescent and LEDs (UV, blue, green, red) |
|  | *Culex pipiens*(Linnaeus) | Incandescent | 570 nm & 660 nm | Kenya | [9] | Incandescent and LEDs (BGR, violet, blue, green, red) |
|  | *Culex ribeirensis* (Forattini & Sallum) | 570 nm & 430 nm | 660 nm | Maranhão, Brazil | [58] | Incandescent and LEDs (UV, blue, green, red) |
|  | *Culiseta melanura* (Coquillett) | Incandescent | 613 nm & 940 nm | Florida, USA | [15] | Incandescent and LEDs (blue, green, yellow, orange, red, IR) |
|  | *Mansonia africana* (Theobald) | Incandescent | 390 nm | Kenya | [9] | Incandescent and LEDs (BGR, violet, blue, green, red) |
|  | *Mansonia titillans* (Walker) | 470 nm & 502 nm | 660 nm & 860 nm | Florida, USA | [66]* | LEDs (blue, green, red, IR) |
|  | *Mansonia amazonensis*(Theobald) |  | Red chemical stick | Peru | [17] | Incandescent and chemical sticks (white, blue, green, yellow, red) |
|  | *Mansonia uniformis* (Theobald) | Incandescent | 660 nm & 390 nm | Kenya | [9] | Incandescent and LEDs (BGR, violet, blue, green, red) |
|  | *Psorophora columbiae* (Dyar & Knab) | 450 nm |  | Florida, USA | [15] | Incandescent and LEDs (blue, green, yellow, orange, red, IR) |
|  | *Uranotaenia apicalis* (Theobald) | Incandescent |  | Peru | [17] | Incandescent and chemical sticks (white, blue, green, yellow, red) |
|  | *Uranotaenia geometrica* (Theobald) | Incandescent |  | Peru | [17] | Incandescent and chemical sticks (white, blue, green, yellow, red) |
|  | *Uranotaenia sapphirina* (Osten Sacken) | Incandescent |  | Florida, USA | [15] | Incandescent and LEDs (blue, green, yellow, orange, red, IR) |
| Phlebotominae | *Evandromyia evandroi*(Costa Lima and Antunes) |  | Incandescent | Maranhão, Brazil | [60] | Incandescent and LEDs (blue, green) |
|  |  | 520 nm | Incandescent | Maranhão, Brazil | [61] | Incandescent and LEDs (blue, green) |
|  |  | 470 nm |  | Maranhão, Brazil | [78] | LEDs (blue, green) |
|  |  | 520 nm |  | Maranhão, Brazil | [63] | Incandescent and LED (green) |
|  | *Evandromyia lenti*(Mangabeira) |  | Incandescent | Maranhão, Brazil | [60] | Incandescent and LEDs (blue, green) |
|  |  | 520 nm |  | Maranhão, Brazil | [63] | Incandescent and LED (green) |
|  | *Evandromyia termitophila* (Martins, Falcão & Silva) |  | Incandescent | Maranhão, Brazil | [60] | Incandescent and LEDs (blue, green) |
|  | *Lutzomyia longipalpis* complex | Incandescent |  | Maranhão, Brazil | [60] | Incandescent and LEDs (blue, green) |
|  |  | 489 nm | White LED | Argentina | [117] | Incandescent and LEDs (white, blue) |
|  |  | 520 nm |  | Maranhão, Brazil | [61] | Incandescent and LEDs (blue, green) |
|  |  | 470 nm |  | Maranhão, Brazil | [78] | LEDs (blue, green) |
|  |  | 520 nm |  | Maranhão, Brazil | [63] | Incandescent and LED (green) |
|  | *Lutzomyia shannoni*(Dyar) | 660 nm | BGR | Florida, USA | [73] | LEDs (BGR, blue, green, red) |
|  | *Lutzomyia vexator*(Coquillett) | BGR | 660 nm | Florida, USA | [73] | LEDs (BGR, blue, green, red) |
|  | *Micropygomyia goiana* (Martins, Falcão & Silva) | 520 nm |  | Maranhão, Brazil | [61] | Incandescent and LEDs (blue, green) |
|  |  | 470 nm |  | Maranhão, Brazil | [78] | LEDs (blue, green) |
|  |  | 520 nm |  | Maranhão, Brazil | [63] | Incandescent and LED (green) |
|  | *Micropygomyia echinatopharynx*(Andrade-Filho) | Incandescent |  | Maranhão, Brazil | [60] | Incandescent and LEDs (blue, green) |
|  | *Micropygomyia trinidadensis*(Newstead) | 520 nm |  | Maranhão, Brazil | [60] | Incandescent and LEDs (blue, green) |
|  | *Migonemyia migonei* (França) | Incandescent | White LED | Argentina | [117] | Incandescent and LEDs (white, blue) |
|  | *Nyssomyia whitmani* (Antunes & Coutinho) | 520 nm | Incandescent | Maranhão, Brazil | [60] | Incandescent and LEDs (blue, green) |
|  |  |  | UV | Argentina | [117] | Incandescent and LEDs (white, blue) |
|  |  | 470 nm | Incandescent | Maranhão, Brazil | [61] | Incandescent and LEDs (blue, green) |
|  |  | 520 nm |  | Maranhão, Brazil | [63] | Incandescent and LED (green) |
|  | *Phlebotomus alexandri* (Sinton) | UV |  | Iraq | [46] | Incandescent and UV |
|  | *Phlebotomus longicuspis* (Nitzulescu) | UV |  | Libya | [121] | Incandescent and UV |
|  | *Phlebotomus papatasi* (Scopoli) | 660 nm | 470 nm & 502 nm | Egypt | [72] | Incandescent and LEDs (blue, green, red) |
|  |  | UV |  | Isreal | [48] | Incandescent and UV |
|  |  | UV |  | Isreal | [51] | Incandescent and UV |
|  | *Sergentomyia africana* (Newstead) | Incandescent | Red chemical stick | Ethiopia | [120] | Incandescent and chemical sticks (green, yellow, red) |
|  | *Sergentomyia antennata* (Newstead) | Incandescent | Yellow chemical stick | Ethiopia | [120] | Incandescent and chemical sticks (green, yellow, red) |
|  | *Sergentomyia clydei* (Sinton) | Incandescent |  | Ethiopia | [120] | Incandescent and chemical sticks (green, yellow, red) |
|  | *Sergentomyia minuta* complex | Incandescent | Red chemical stick | Ethiopia | [120] | Incandescent and chemical sticks (green, yellow, red) |
|  | *Sergentomyia schwtezi* (Adler, Theodor & Parrot) | Incandescent | Green & yellow chemical sticks | Ethiopia | [120] | Incandescent and chemical sticks (green, yellow, red) |
|  | *Sergentomyia squamipleuris* (Newstead) | Incandescent |  | Ethiopia | [120] | Incandescent and chemical sticks (green, yellow, red) |

Wavelength preferences/biases of various species of Ceratopogonidae, Culicidae, and Phlebotominae in experiments comparing catches between lights of different wavelengths. What constitutes a preference/bias was determined on a case-by-case basis. Species with inconclusive preferences/biases are not listed here. Colour is used in cases where dominant wavelength is not specified. * Data from resting boxes not included in table.

**References**

1. Tchouassi DP, Sang R, Sole CL, Bastos AD, Cohnstaedt LW, Torto B. Trapping of Rift Valley Fever (RVF) vectors using Light Emitting Diode (LED) CDC traps in two arboviral disease hot spots in Kenya. Parasites and Vectors. 2012;5:94.
2. Burkett DA, Butler JF, Kline DL. Field evaluation of colored light-emitting diodes as attractants for woodland mosquitoes and other Diptera in north central Florida. Journal of the American Mosquito Control Association. 1998;14:186-195.
3. Rogers E, Sholdt LL, Falcón R. Effects of incorporating chemical light sources in CDC traps: differences in the capture rates of neotropical *Culex*, *Anopheles* and *Uranotaenia* (Diptera: Culicidae). The Pan-Pacific Entomologist. 1993;69:141-148.
4. Bishop AL, Worrall R, Spohr LJ, McKenzie HJ, Barchia IM. Response of *Culicoides* spp. (Diptera: Ceratopogonidae) to light‐emitting diodes. Australian Journal of Entomology. 2004;43:184-188.
5. Bishop AL, Bellis GA, McKenzie HJ, Spohr LJ, Worrall RJ, Harris AM, Melville L. Light trapping of biting midges *Culicoides* spp. (Diptera: Ceratopogonidae) with green light‐emitting diodes. Australian Journal of Entomology. 2006;45:202-205.
6. Baik LS, Nave C, Au DD, Guda T, Chevez JA, Ray A, Holmes TC. Circadian regulation of light-evoked attraction and avoidance behaviors in daytime-versus nighttime-biting mosquitoes. Current Biology. 2020;30:3252-3259.
7. Burkett DA, Knight R, Dennett JA, Sherwood V, Rowton E, Coleman RE. Impact of phlebotomine sand flies on US military operations at Tallil Air Base, Iraq: 3. Evaluation of surveillance devices for the collection of adult sand flies. Journal of Medical Entomology. 2007;44:381-384.
8. Kline DL, Hogsette JA, Müller GC. Comparison of various configurations of CDC‐type traps for the collection of *Phlebotomus papatasi* Scopoli in southern Israel. Journal of Vector Ecology. 2011;36:S212-S218.
9. Obenauer PJ, Abdel-Dayem MS, Stoops CA, Villinski JT, Tageldin R, Fahmy NT, Diclaro JW, Bolay F. Field responses of *Anopheles gambiae* complex (Diptera: Culicidae) in Liberia using yeast-generated carbon dioxide and synthetic lure-baited light traps. Journal of Medical Entomology. 2013;50:863-870.
10. Müller GC, Hogsette JA, Kline DL, Beier JC, Revay EE, Xue RD. Response of the sand fly *Phlebotomus papatasi* to visual, physical and chemical attraction features in the field. Acta Tropica. 2015;141:32-36.
11. Silva JS, Souto Couri M, de Leão Giupponi AP, Alencar J. Mosquito fauna of the Guapiaçu Ecological Reserve, Cachoeiras de Macacu, Rio de Janeiro, Brazil, collected under the influence of different color CDC light traps. Journal of Vector Ecology. 2014;39:384-394.
12. Silva FS, Brito JM, Costa-Neta BM. Field evaluation of light-emitting diode as attractant for blood-sucking midges of the genus *Culicoides Latreille* (Diptera: Culicomorpha, Ceratopogonidae) in the Brazilian savanna. Entomological News. 2015;125:1-6.
13. Silva FS, Brito JM, Costa Neta BM, Lobo SEPD. Evaluation of light emitting diodes as attractant for sandflies (Diptera: Psychodidae: Phlebotominae) in northeastern Brazil. Memórias do Instituto Oswaldo Cruz. 2015;110:801-803.
14. Silva FS, da Silva AA, Rebêlo JMM. An evaluation of light-emitting diode (LED) traps at capturing phlebotomine sand flies (Diptera: Psychodidae) in a livestock area in Brazil. Journal of Medical Entomology. 2016;53:634-638.
15. Costa-Neta BM, da Silva AA, Brito JM, Moraes JLP, Rebêlo JMM, Silva FS. Light-emitting diode (LED) traps improve the light-trapping of anopheline mosquitoes. Journal of Medical Entomology. 2017;54:1699-1703.
16. da Silva AA, Rebêlo JMM, Carneiro BF, Castro MPP, de Sousa de Almeida M, Ponte IS, Aguiar JVC, Silva FS. Exploiting the synergistic effect of kairomones and light-emitting diodes on the attraction of phlebotomine sand flies to light traps in Brazil. Journal of Medical Entomology. 2019;56:1441-1445.
17. Bentley MT, Kaufman PE, Kline DL, Hogsette JA. Response of adult mosquitoes to light-emitting diodes placed in resting boxes and in the field. Journal of the American Mosquito Control Association. 2009;25:285-291.
18. Hope A, Gubbins S, Sanders C, Denison E, Barber J, Stubbins F, Baylis M, Carpenter S. A comparison of commercial light-emitting diode baited suction traps for surveillance of *Culicoides* in northern Europe. Parasites and Vectors. 2015;8:239.
19. González M, Alarcón-Elbal PM, Valle-Mora J, Goldarazena A. Comparison of different light sources for trapping *Culicoides* biting midges, mosquitoes and other dipterans. Veterinary Parasitology. 2016;226:44-49.
20. Snyder D, Cernicchiaro N, Cohnstaedt LW. Sugar‐feeding status alters biting midge photoattraction. Medical and Veterinary Entomology. 2016;30:31-38.
21. Venter GJ, Boikanyo SN, De Beer CJ. The efficiency of light‐emitting diode suction traps for the collection of South African livestock associated *Culicoides* species. Medical and Veterinary Entomology. 2018;32:509-514.
22. Hoel DF, Butler JF, Fawaz EY, Watany N, El-Hossary SS, Villinski J. Response of phlebotomine sand flies to light-emitting diode-modified light traps in southern Egypt. Journal of Vector Ecology. 2007;32:302-308.
23. Mann RS, Kaufman PE, Butler JF. *Lutzomyia* spp. (Diptera: Psychodidae) response to olfactory attractant-and light emitting diode-modified Mosquito Magnet X (MM-X) traps. Journal of Medical Entomology. 2009;46:1052-1061.
24. Costa-Neta BM, Lima-Neto AR, da Silva AA, Brito JM, Aguiar JVC, Ponte IS, Silva FS. Centers for Disease Control-type light traps equipped with high-intensity light-emitting diodes as light sources for monitoring *Anopheles* mosquitoes. Acta tropica. 2018;183:61-63.
25. Lima-Neto AR, Costa-Neta BM, da Silva AA, Brito JM, Aguiar JV, Ponte IS, Silva FS. The effect of luminous intensity on the attraction of phlebotomine sand flies to light traps. Journal of Medical Entomology. 2018;55:731-734.
26. Fernández MS, Martínez MF, Pérez AA, Santini MS, Gould IT, Salomón OD. Performance of light‐emitting diode traps for collecting sand flies in entomological surveys in Argentina. Journal of Vector Ecology. 2015;40:373-378.
27. Grogan WL, Spinelli GR, Phillips RA, Woodward DL. The male of *Culicoides reevesi* Wirth, with a redescription of the female and new seasonal activity, distribution, and biting records (Diptera: Ceratopogonidae). Western North American Naturalist. 2004;64:433-438.
28. Harrup LE, Laban S, Purse BV, Reddy YK, Reddy YN, Byregowda SM, Kumar N, Purushotham KM, Kowalli S, Prasad M, Prasad G. DNA barcoding and surveillance sampling strategies for *Culicoides* biting midges (Diptera: Ceratopogonidae) in southern India. Parasites and Vectors. 2016;9:461.
29. Kirstein OD, Faiman R, Gebreselassie A, Hailu A, Gebre-Michael T, Warburg A. Attraction of Ethiopian phlebotomine sand flies (Diptera: Psychodidae) to light and sugar-yeast mixtures (CO 2). Parasites & Vectors. 2013;6:341.
30. Obenauer PJ, Annajar BB, Hanafi HA, Abdel-Dayem MS, El-Hossary SS, Villinski J. Efficacy of light and nonlighted carbon dioxide–baited traps for adult sand fly (Diptera: Psychodidae) surveillance in three counties of Mesrata, Libya. Journal of the American Mosquito Control Association. 2012;28:179-183.
31. Venter GJ, Hermanides KG. Comparison of black and white light for collecting *Culicoides imicola* and other livestock-associated *Culicoides* species in South Africa. Veterinary Parasitology. 2006;142:383-385.
